# Tetra­ethyl­ammonium (2,2′-bipyridine)tetra­cyanidocobaltate(III) sesquihydrate acetonitrile solvate

**DOI:** 10.1107/S1600536810011311

**Published:** 2010-03-31

**Authors:** Ganna Lyubartseva, Sean Parkin

**Affiliations:** a Department of Chemistry and Physics, College of Science and Technology, Southern Arkansas University, Magnolia, AR 71753, USA; b Department of Chemistry, University of Kentucky, Lexington, KY 40506, USA

## Abstract

The title complex, (C_8_H_20_N)[Co(CN)_4_(C_10_H_8_N_2_)]·CH_3_CN·1.5H_2_O, consists of tetra­ethyl ammonium cations, mononuclear [Co^III^bpy(CN)_4_]^−^ anions and uncoordinated water and acetonitrile mol­ecules. The Co^III^ atom is six-coordinated by two 2,2′-bipyridine (bpy) N atoms and four cyanide C atoms in a distorted octa­hedral geometry. The acute bite angle of the chelating bpy [82.28 (8)°] is the main factor accounting for this distortion. In addition, the tetra­ethyl­ammonium cation is significantly disordered [occupancy ratio 0.611 (3):0.389 (3)]. The presence of water mol­ecules, one of which is disordered over two positions about an inversion center, results in the formation of a network of O—H⋯N hydrogen bonds involving the cyanide N atoms.

## Related literature

For the starting complex [Co(bpy)_3_]Cl_2_·2H_2_O·CH_3_CH_2_OH, see: Szalda *et al.* (1983 ▶). For a similar building block with a tetra­phenyl­phospho­nium cation and chromium(III), and a nuclearity controlled cyanide-bridged bimetallic compound, see: Toma *et al.* (2004 ▶); with a potassium cation and tetra­phenyl­arsonium cation and iron(III), and cyanide-bridged heterobimetallic complexes, see: Toma *et al.* (2007 ▶); with a tetra­phenyl­phospho­nium cation and iron(III) and ribbon-like ferromagnetic cyano-bridged chains, see: Lescoueezec *et al.* (2002 ▶). For potential applications of bimetallic clusters as catalysts, see: Darensbourg & Phelps (2004 ▶), as room temperature magnets, see: Mallah *et al.* (1993 ▶); Garde *et al.* (2002 ▶); Holmes & Girolami (1999 ▶) and as single-mol­ecule magnets, see: Sokol *et al.* (2002 ▶). For attempts to make a similar building block with nickel(II), see: Lyubartseva & Parkin (2009 ▶).
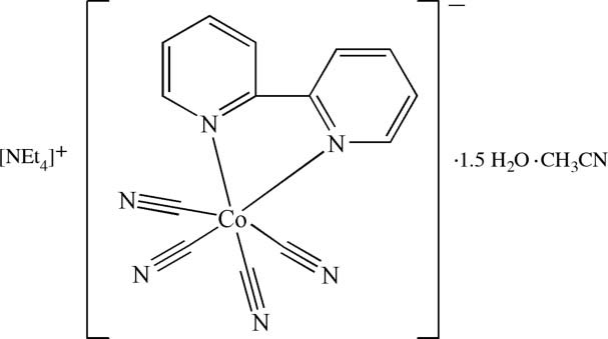


## Experimental

### 

#### Crystal data

(C_8_H_20_N)[Co(CN)_4_(C_10_H_8_N_2_)]·C_2_H_3_N·1.5H_2_O
*M* *_r_* = 517.52
Monoclinic, 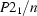

*a* = 10.1591 (1) Å
*b* = 23.4015 (3) Å
*c* = 11.3422 (2) Å
β = 97.3080 (5)°
*V* = 2674.57 (6) Å^3^
*Z* = 4
Mo *K*α radiation
μ = 0.68 mm^−1^
*T* = 90 K
0.30 × 0.27 × 0.25 mm

#### Data collection

Nonius KappaCCD diffractometer
Absorption correction: multi-scan (*SCALEPACK*; Otwinowski & Minor, 1997 ▶) *T*_min_ = 0.709, *T*_max_ = 0.849
28623 measured reflections
4704 independent reflections
3689 reflections with *I* > 2σ(*I*)
*R*_int_ = 0.048

#### Refinement

*R*[*F* ^2^ > 2σ(*F* ^2^)] = 0.041
*wR*(*F* ^2^) = 0.108
*S* = 1.05
4704 reflections
378 parameters
60 restraints
H atoms treated by a mixture of independent and constrained refinement
Δρ_max_ = 0.69 e Å^−3^
Δρ_min_ = −0.33 e Å^−3^

### 

Data collection: *COLLECT* (Nonius, 1998 ▶); cell refinement: *SCALEPACK* (Otwinowski & Minor, 1997 ▶); data reduction: *DENZO-SMN* (Otwinowski & Minor, 1997 ▶); program(s) used to solve structure: *SHELXS97* (Sheldrick, 2008 ▶); program(s) used to refine structure: *SHELXL97* (Sheldrick, 2008 ▶); molecular graphics: *XP* in *SHELXTL* (Sheldrick, 2008 ▶); software used to prepare material for publication: *SHELXL97* and local procedures.

## Supplementary Material

Crystal structure: contains datablocks global, I. DOI: 10.1107/S1600536810011311/kp2254sup1.cif

Structure factors: contains datablocks I. DOI: 10.1107/S1600536810011311/kp2254Isup2.hkl

Additional supplementary materials:  crystallographic information; 3D view; checkCIF report

## Figures and Tables

**Table 1:** Hydrogen-bond geometry (Å, °)

*D*—H⋯*A*	*D*—H	H⋯*A*	*D*⋯*A*	*D*—H⋯*A*
O1*W*—H1*W*1⋯N5^i^	0.83 (2)	2.10 (2)	2.925 (3)	176 (3)
O1*W*—H2*W*1⋯N4	0.82 (2)	2.08 (2)	2.899 (3)	174 (3)
O2*W*—H1*W*2⋯N3	0.84 (2)	1.83 (2)	2.665 (5)	172 (7)
O2*W*—H2*W*2⋯N3^ii^	0.84 (2)	1.92 (2)	2.740 (5)	165 (7)

Symmetry codes: (i) 
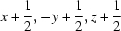
; (ii) 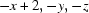.

**Fig. 1.**
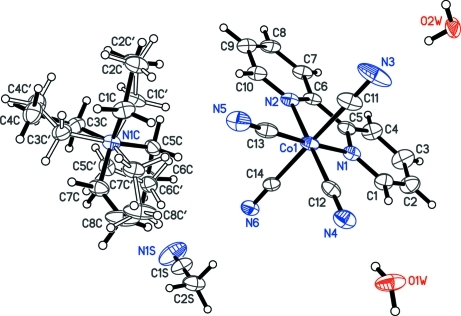
View of the title compound with the atom numbering scheme. Displacement ellipsoids are drawn at the 50% probability level.

## supplementary crystallographic information

### Comment

Self-assembly of cyano-linked metal complexes draws considerable interest as a strategy for developing new bimetallic clusters. Typically one needs a stable cyanometallate anion as a ligand for fully or partially solvated metal ions. Hexacyanometallate anion [M(CN)_6_]^3-^ is very useful but it often gives three dimensional Prussian Blue analogs. If the metal ions are partially blocked with a bidentate ligand it is possible to control the nuclearity of the bimetallic clusters which have potential application as catalysts, see Darensbourg *et al.* (2004); room temperature magnets, see Mallah *et al.* (1993), Garde *et al.* (2002) and Holmes & Girolami (1999); or single-molecule magnets, see Sokol *et al.* (2002). Here we report a new building block [(Co^III^bpy(CN)_4_]^-^- which can be applied to make new bimetallic clusters.

The structure consists of tetraethyl ammonium cations, mononuclear [Co^III^bpy(CN)_4_]^-^ anions, uncoordinated water and acetonitrile molecules. The cobalt (III) atom is six-coordinate with two bpy-nitrogen atoms and four cyanide-carbon atoms in a distorted octahedral geometry (Fig.1). The acute bite angle of the chelating bpy [82.28 (8)°] is the main factor accounting for this distortion from the ideal geometry. The values of the Co^III^-nitrogen bond lengths are shorter than bond lengths in the similar chromium (III) anionic complex reported by Toma *et al.* (2004). The cobalt—carbon—nitrogen angles for the terminally bound cyanide ligands are quasilinear [178.5 (2)–179.5 (3)°]. The values of the cyanide bonds vary in the range 1.147 (4)–1.159 (3) Å. The presence of terminally bound cyanide groups in the structure is consistent with the presence of cyanide stretching vibrations at 2133(*m*)cm^-1^. Also, the tetraethyl ammonium cationic part is significantly disordered. Presence of water molecules, one of which is disordered over two positions about an inversion centre,  results in the formation of a network of O—H···N hydrogen bonds involving nitrogen atoms of cyano ligands.

### Experimental

2,2'-bipyridine and tetraethylammonium cyanide were purchased from Sigma-Aldrich and used as received. The starting complex [Co(bpy)_3_]Cl_2_.2H_2_O.CH_3_CH_2_OH was synthesized according to the previously published procedure by Szalda *et al.*(1983). [Co(bpy)_3_]Cl_2_.2H_2_0.CH_3_CH_2_OH (680 mg, 1 mmol) was dissolved in 20 ml 1:1 mixture of methanol and acetonitrile. Tetraethylammonium cyanide (468 mg, 3 mmol) was dissolved in 10 ml wet acetonitrile. The cyanide solution was added dropwise to metal solution with slow stirring. Once the addition was complete, the resulting solution was filtered and solvent was evaporated slowly. Yellow coloured, analytically pure monoclinic crystals were obtained after one week and found to be [tetraethylammonium][(2-(pyridin-2-yl)pyridine) tetracyanocobaltate (III)]. 2H_2_O.CH_3_CN (198 mg, 37.6% yield). Elemental analysis calculated for CoC_24_H_35_N_8_O_2_: C 54.75, H 6.70, N 21.28; found C 55.55, H 6.49, N 20.43. IR(cm^-1^) 3496, 2997, 2133, 1578, 1557, 1453, 1412, 1313, 1250, 1185, 1083, 997, 791, 773,757,653, 620, 403.

### Refinement

H atoms on the anion, cation, and acetonitrile were found in difference Fourier maps and later placed in idealized positions with constrained distances of 0.98 Å (RCH_3_), 0.99 Å (R_2_CH_2_), and 0.95 Å (C_Ar_H), and with U_iso_(H) values set to either 1.2U_eq_ or 1.5U_eq_ (RCH_3_) of the attached atom. The H atoms attached to O1w were found in a difference map. The partial occupancy O2w is within H-bonding distance of N3, so it was assumed that one of the hydrogen atoms of this water would be found between O2w and N3. Indeed, a pair of plausible hydrogen atoms were found in a difference map, with one of them between O2w and N3, in spite of the partial occupancy. The water hydrogens were constrained to distances of 0.84 Å and assigned U_iso_ of 1.5U_eq_ (O).

To ensure chemically sensible and physically reasonable structural parameters for the disordered cation, the SHELXL97 SAME and DELU restraints were used along with the EADP constraint. Water molecule geometry was restrained using the SHELXL97 DFIX command.

### Crystal data

**Table:**

(C_8_H_20_N)[Co(CN)_4_(C_10_H_8_N_2_)]·C_2_H_3_N·1.5H_2_O	*F*(000) = 1092
*M**_r_* = 517.52	*D*_x_ = 1.285 Mg m^−^^3^
Monoclinic, *P*2_1_/*n*	Mo *K*α radiation, λ = 0.71073 Å
Hall symbol: -P 2yn	Cell parameters from 6273 reflections
*a* = 10.1591 (1) Å	θ = 1.0–27.5°
*b* = 23.4015 (3) Å	µ = 0.68 mm^−^^1^
*c* = 11.3422 (2) Å	*T* = 90 K
β = 97.3080 (5)°	Block, yellow
*V* = 2674.57 (6) Å^3^	0.30 × 0.27 × 0.25 mm
*Z* = 4	

### Data collection

**Table:**

Nonius KappaCCD diffractometer	4704 independent reflections
Radiation source: fine-focus sealed tube	3689 reflections with *I* > 2σ(*I*)
graphite	*R*_int_ = 0.048
Detector resolution: 9.1 pixels mm^-1^	θ_max_ = 25.0°, θ_min_ = 2.0°
ω scans at fixed χ = 55°	*h* = −12→12
Absorption correction: multi-scan (*SCALEPACK*; Otwinowski & Minor, 1997)	*k* = −27→27
*T*_min_ = 0.709, *T*_max_ = 0.849	*l* = −13→13
28623 measured reflections	

### Refinement

**Table:**

Refinement on *F*^2^	Primary atom site location: structure-invariant direct methods
Least-squares matrix: full	Secondary atom site location: difference Fourier map
*R*[*F*^2^ > 2σ(*F*^2^)] = 0.041	Hydrogen site location: inferred from neighbouring sites
*wR*(*F*^2^) = 0.108	H atoms treated by a mixture of independent and constrained refinement
*S* = 1.05	*w* = 1/[σ^2^(*F*_o_^2^) + (0.0481*P*)^2^ + 2.6305*P*] where *P* = (*F*_o_^2^ + 2*F*_c_^2^)/3
4704 reflections	(Δ/σ)_max_ = 0.001
378 parameters	Δρ_max_ = 0.69 e Å^−^^3^
60 restraints	Δρ_min_ = −0.33 e Å^−^^3^

### Special details

**Table:**

Geometry. All esds (except the esd in the dihedral angle between two l.s. planes) are estimated using the full covariance matrix. The cell esds are taken into account individually in the estimation of esds in distances, angles and torsion angles; correlations between esds in cell parameters are only used when they are defined by crystal symmetry. An approximate (isotropic) treatment of cell esds is used for estimating esds involving l.s. planes.
Refinement. Refinement of *F*^2^ against ALL reflections. The weighted *R*-factor wR and goodness of fit *S* are based on *F*^2^, conventional *R*-factors *R* are based on *F*, with *F* set to zero for negative *F*^2^. The threshold expression of *F*^2^ > 2σ(*F*^2^) is used only for calculating *R*-factors(gt) etc. and is not relevant to the choice of reflections for refinement. *R*-factors based on *F*^2^ are statistically about twice as large as those based on *F*, and *R*- factors based on ALL data will be even larger.

### Fractional atomic coordinates and isotropic or equivalent isotropic displacement parameters (Å^2^)

**Table:**

	*x*	*y*	*z*	*U*_iso_*/*U*_eq_	Occ. (<1)
Co1	0.66787 (3)	0.093308 (13)	0.27408 (3)	0.02317 (12)	
N1	0.7419 (2)	0.05486 (8)	0.42108 (19)	0.0238 (5)	
N2	0.5597 (2)	0.02349 (8)	0.25393 (18)	0.0240 (5)	
C1	0.8401 (3)	0.07455 (11)	0.5019 (2)	0.0292 (6)	
H1	0.8782	0.1108	0.4895	0.035*	
C2	0.8872 (3)	0.04358 (12)	0.6025 (3)	0.0383 (7)	
H2	0.9566	0.0584	0.6582	0.046*	
C3	0.8320 (3)	−0.00906 (13)	0.6209 (3)	0.0431 (8)	
H3	0.8626	−0.0308	0.6896	0.052*	
C4	0.7314 (3)	−0.02991 (12)	0.5380 (2)	0.0350 (7)	
H4	0.6926	−0.0661	0.5491	0.042*	
C5	0.6881 (3)	0.00296 (10)	0.4384 (2)	0.0254 (6)	
C6	0.5839 (3)	−0.01476 (10)	0.3436 (2)	0.0239 (6)	
C7	0.5152 (3)	−0.06600 (11)	0.3423 (2)	0.0287 (6)	
H7	0.5339	−0.0925	0.4057	0.034*	
C8	0.4195 (3)	−0.07813 (11)	0.2483 (2)	0.0320 (6)	
H8	0.3712	−0.1129	0.2463	0.038*	
C9	0.3946 (3)	−0.03900 (11)	0.1567 (2)	0.0321 (6)	
H9	0.3293	−0.0465	0.0909	0.039*	
C10	0.4668 (3)	0.01138 (11)	0.1630 (2)	0.0293 (6)	
H10	0.4497	0.0383	0.1002	0.035*	
N3	0.8813 (3)	0.03848 (11)	0.1447 (3)	0.0600 (9)	
C11	0.8014 (3)	0.05929 (11)	0.1935 (3)	0.0350 (7)	
N4	0.8354 (2)	0.19977 (9)	0.3179 (2)	0.0337 (5)	
C12	0.7721 (3)	0.15893 (11)	0.3010 (2)	0.0263 (6)	
N5	0.5526 (3)	0.14664 (10)	0.0403 (2)	0.0375 (6)	
C13	0.5958 (3)	0.12650 (11)	0.1303 (2)	0.0293 (6)	
N6	0.4571 (2)	0.15090 (9)	0.4029 (2)	0.0281 (5)	
C14	0.5359 (2)	0.12964 (10)	0.3530 (2)	0.0224 (5)	
N1C	0.1397 (2)	0.17482 (9)	0.03158 (18)	0.0240 (5)	
C1C	0.2239 (5)	0.16359 (19)	−0.0684 (4)	0.0317 (11)	0.611 (3)
H1C1	0.1910	0.1883	−0.1368	0.038*	0.611 (3)
H1C2	0.3164	0.1752	−0.0408	0.038*	0.611 (3)
C2C	0.225 (3)	0.1027 (8)	−0.1111 (16)	0.0409 (18)	0.611 (3)
H2C1	0.2810	0.0997	−0.1750	0.061*	0.611 (3)
H2C2	0.1343	0.0909	−0.1410	0.061*	0.611 (3)
H2C3	0.2602	0.0778	−0.0450	0.061*	0.611 (3)
C3C	0.0006 (4)	0.1547 (2)	0.0034 (4)	0.0306 (11)	0.611 (3)
H3C1	−0.0495	0.1659	0.0691	0.037*	0.611 (3)
H3C2	0.0007	0.1124	−0.0005	0.037*	0.611 (3)
C4C	−0.0712 (10)	0.1778 (4)	−0.1124 (10)	0.040 (2)	0.611 (3)
H4C1	−0.1620	0.1627	−0.1244	0.060*	0.611 (3)
H4C2	−0.0241	0.1658	−0.1785	0.060*	0.611 (3)
H4C3	−0.0739	0.2196	−0.1090	0.060*	0.611 (3)
C5C	0.2059 (4)	0.14187 (17)	0.1426 (4)	0.0271 (10)	0.611 (3)
H5C1	0.2023	0.1005	0.1246	0.033*	0.611 (3)
H5C2	0.3007	0.1530	0.1573	0.033*	0.611 (3)
C6C	0.1447 (19)	0.1518 (9)	0.2535 (7)	0.029 (2)	0.611 (3)
H6C1	0.1928	0.1297	0.3187	0.044*	0.611 (3)
H6C2	0.0517	0.1396	0.2412	0.044*	0.611 (3)
H6C3	0.1495	0.1925	0.2735	0.044*	0.611 (3)
C7C	0.1441 (5)	0.23881 (17)	0.0576 (4)	0.0310 (11)	0.611 (3)
H7C1	0.1056	0.2565	−0.0183	0.037*	0.611 (3)
H7C2	0.0770	0.2443	0.1127	0.037*	0.611 (3)
C8C	0.2393 (7)	0.2726 (3)	0.0975 (11)	0.049 (2)	0.611 (3)
H8C1	0.2046	0.3114	0.1036	0.074*	0.611 (3)
H8C2	0.3069	0.2725	0.0431	0.074*	0.611 (3)
H8C3	0.2789	0.2596	0.1762	0.074*	0.611 (3)
C1C'	0.1829 (7)	0.1151 (3)	0.0039 (6)	0.0313 (17)	0.389 (3)
H1C3	0.2515	0.1028	0.0687	0.038*	0.389 (3)
H1C4	0.1058	0.0893	0.0049	0.038*	0.389 (3)
C2C'	0.237 (4)	0.1069 (13)	−0.112 (3)	0.0409 (18)	0.389 (3)
H2C4	0.2643	0.0670	−0.1196	0.061*	0.389 (3)
H2C5	0.3141	0.1319	−0.1150	0.061*	0.389 (3)
H2C6	0.1685	0.1164	−0.1781	0.061*	0.389 (3)
C3C'	0.0367 (7)	0.1966 (3)	−0.0737 (6)	0.0302 (16)	0.389 (3)
H3C3	0.0068	0.2353	−0.0539	0.036*	0.389 (3)
H3C4	0.0820	0.2000	−0.1457	0.036*	0.389 (3)
C4C'	−0.0849 (16)	0.1587 (7)	−0.1027 (18)	0.040 (2)	0.389 (3)
H4C4	−0.1428	0.1749	−0.1701	0.060*	0.389 (3)
H4C5	−0.1333	0.1566	−0.0334	0.060*	0.389 (3)
H4C6	−0.0569	0.1203	−0.1232	0.060*	0.389 (3)
C5C'	0.0653 (6)	0.1730 (3)	0.1398 (5)	0.0225 (14)	0.389 (3)
H5C3	0.0202	0.2101	0.1467	0.027*	0.389 (3)
H5C4	−0.0039	0.1431	0.1273	0.027*	0.389 (3)
C6C'	0.152 (3)	0.1611 (15)	0.2544 (12)	0.029 (2)	0.389 (3)
H6C4	0.0982	0.1601	0.3199	0.044*	0.389 (3)
H6C5	0.2194	0.1913	0.2691	0.044*	0.389 (3)
H6C6	0.1967	0.1241	0.2491	0.044*	0.389 (3)
C7C'	0.2528 (6)	0.2158 (3)	0.0447 (6)	0.0274 (16)	0.389 (3)
H7C3	0.3307	0.1902	0.0616	0.033*	0.389 (3)
H7C4	0.2551	0.2291	−0.0378	0.033*	0.389 (3)
C8C'	0.2884 (13)	0.2594 (5)	0.1049 (17)	0.049 (2)	0.389 (3)
H8C4	0.3714	0.2741	0.0811	0.074*	0.389 (3)
H8C5	0.3021	0.2494	0.1895	0.074*	0.389 (3)
H8C6	0.2194	0.2888	0.0911	0.074*	0.389 (3)
N1S	0.0766 (3)	0.15711 (15)	0.6193 (3)	0.0686 (10)	
C1S	0.1527 (3)	0.18783 (14)	0.5901 (3)	0.0407 (7)	
C2S	0.2493 (3)	0.22731 (12)	0.5543 (3)	0.0404 (7)	
H2S1	0.2060	0.2531	0.4933	0.061*	
H2S2	0.3197	0.2060	0.5220	0.061*	
H2S3	0.2879	0.2496	0.6233	0.061*	
O1W	1.0262 (2)	0.29166 (9)	0.31420 (19)	0.0423 (5)	
H1W1	1.034 (3)	0.3105 (13)	0.376 (2)	0.063*	
H2W1	0.969 (3)	0.2672 (12)	0.318 (3)	0.063*	
O2W	1.1073 (4)	−0.0035 (2)	0.0843 (4)	0.0494 (12)	0.50
H1W2	1.032 (4)	0.008 (3)	0.098 (6)	0.074*	0.50
H2W2	1.098 (7)	−0.010 (3)	0.011 (2)	0.074*	0.50

### Atomic displacement parameters (Å^2^)

**Table:**

	*U*^11^	*U*^22^	*U*^33^	*U*^12^	*U*^13^	*U*^23^
Co1	0.0327 (2)	0.01501 (19)	0.0242 (2)	−0.00140 (14)	0.01285 (15)	−0.00014 (14)
N1	0.0272 (11)	0.0178 (10)	0.0289 (12)	−0.0035 (9)	0.0129 (9)	−0.0010 (9)
N2	0.0318 (12)	0.0179 (10)	0.0246 (12)	−0.0017 (9)	0.0120 (9)	−0.0022 (9)
C1	0.0329 (15)	0.0223 (13)	0.0335 (15)	−0.0050 (11)	0.0084 (12)	0.0009 (12)
C2	0.0467 (17)	0.0315 (16)	0.0353 (17)	−0.0054 (13)	0.0001 (14)	0.0007 (13)
C3	0.059 (2)	0.0351 (17)	0.0338 (17)	−0.0089 (15)	0.0020 (15)	0.0095 (14)
C4	0.0479 (17)	0.0244 (14)	0.0336 (16)	−0.0089 (13)	0.0081 (13)	0.0046 (12)
C5	0.0329 (14)	0.0190 (13)	0.0265 (14)	−0.0009 (11)	0.0126 (11)	−0.0009 (11)
C6	0.0332 (14)	0.0179 (12)	0.0231 (13)	−0.0008 (11)	0.0133 (11)	−0.0028 (10)
C7	0.0417 (16)	0.0185 (13)	0.0289 (15)	−0.0041 (11)	0.0162 (12)	−0.0008 (11)
C8	0.0396 (16)	0.0212 (13)	0.0376 (16)	−0.0090 (12)	0.0149 (13)	−0.0090 (12)
C9	0.0389 (16)	0.0281 (14)	0.0302 (15)	−0.0043 (12)	0.0076 (12)	−0.0081 (12)
C10	0.0416 (16)	0.0222 (14)	0.0253 (14)	0.0005 (12)	0.0094 (12)	−0.0015 (11)
N3	0.086 (2)	0.0285 (14)	0.078 (2)	0.0051 (14)	0.0623 (19)	0.0012 (14)
C11	0.0521 (18)	0.0173 (14)	0.0405 (17)	−0.0043 (12)	0.0255 (14)	0.0019 (12)
N4	0.0389 (13)	0.0227 (12)	0.0425 (14)	−0.0040 (11)	0.0167 (11)	0.0010 (10)
C12	0.0306 (14)	0.0215 (14)	0.0297 (14)	0.0042 (12)	0.0153 (11)	0.0035 (11)
N5	0.0548 (16)	0.0277 (13)	0.0315 (14)	−0.0001 (11)	0.0113 (12)	0.0055 (11)
C13	0.0422 (16)	0.0190 (13)	0.0299 (16)	−0.0052 (12)	0.0166 (13)	−0.0024 (12)
N6	0.0300 (12)	0.0221 (11)	0.0337 (13)	−0.0004 (9)	0.0100 (10)	−0.0031 (10)
C14	0.0287 (14)	0.0161 (12)	0.0227 (13)	−0.0052 (10)	0.0048 (11)	0.0003 (10)
N1C	0.0295 (11)	0.0203 (11)	0.0236 (11)	−0.0016 (9)	0.0087 (9)	0.0000 (9)
C1C	0.041 (3)	0.035 (3)	0.022 (2)	−0.007 (2)	0.0130 (19)	−0.0034 (19)
C2C	0.048 (4)	0.038 (3)	0.0396 (18)	0.006 (3)	0.015 (2)	−0.0112 (17)
C3C	0.028 (2)	0.036 (3)	0.029 (2)	−0.0050 (19)	0.0056 (18)	−0.006 (2)
C4C	0.042 (3)	0.049 (7)	0.028 (2)	0.003 (4)	0.000 (2)	0.002 (4)
C5C	0.031 (2)	0.021 (2)	0.028 (2)	0.0054 (18)	0.0038 (18)	0.0008 (18)
C6C	0.038 (2)	0.021 (7)	0.0286 (15)	0.004 (3)	0.0031 (14)	0.0039 (17)
C7C	0.046 (3)	0.020 (2)	0.027 (2)	0.0013 (19)	0.007 (2)	0.0004 (18)
C8C	0.080 (6)	0.029 (4)	0.048 (3)	−0.016 (4)	0.043 (5)	−0.016 (3)
C1C'	0.037 (4)	0.015 (3)	0.044 (4)	0.002 (3)	0.015 (3)	−0.006 (3)
C2C'	0.048 (4)	0.038 (3)	0.0396 (18)	0.006 (3)	0.015 (2)	−0.0112 (17)
C3C'	0.039 (4)	0.029 (4)	0.022 (3)	0.002 (3)	0.003 (3)	−0.001 (3)
C4C'	0.042 (3)	0.049 (7)	0.028 (2)	0.003 (4)	0.000 (2)	0.002 (4)
C5C'	0.022 (3)	0.021 (3)	0.027 (3)	0.000 (3)	0.011 (3)	0.006 (3)
C6C'	0.038 (2)	0.021 (7)	0.0286 (15)	0.004 (3)	0.0031 (14)	0.0039 (17)
C7C'	0.031 (4)	0.021 (3)	0.033 (4)	−0.007 (3)	0.012 (3)	−0.003 (3)
C8C'	0.080 (6)	0.029 (4)	0.048 (3)	−0.016 (4)	0.043 (5)	−0.016 (3)
N1S	0.078 (2)	0.082 (2)	0.0462 (18)	−0.045 (2)	0.0083 (16)	−0.0021 (16)
C1S	0.0477 (18)	0.0448 (18)	0.0296 (16)	−0.0135 (15)	0.0054 (14)	−0.0090 (14)
C2S	0.0436 (17)	0.0330 (16)	0.0486 (19)	−0.0068 (13)	0.0212 (15)	−0.0123 (14)
O1W	0.0548 (14)	0.0344 (12)	0.0429 (13)	−0.0221 (10)	0.0265 (11)	−0.0162 (10)
O2W	0.033 (2)	0.078 (3)	0.035 (2)	0.018 (2)	−0.0045 (19)	−0.015 (2)

### Geometric parameters (Å, °)

**Table:**

Co1—C12	1.868 (3)	C4C—H4C1	0.9800
Co1—C13	1.869 (3)	C4C—H4C2	0.9800
Co1—C11	1.904 (3)	C4C—H4C3	0.9800
Co1—C14	1.904 (3)	C5C—C6C	1.490 (14)
Co1—N1	1.959 (2)	C5C—H5C1	0.9900
Co1—N2	1.966 (2)	C5C—H5C2	0.9900
N1—C1	1.347 (3)	C6C—H6C1	0.9800
N1—C5	1.356 (3)	C6C—H6C2	0.9800
N2—C10	1.337 (3)	C6C—H6C3	0.9800
N2—C6	1.354 (3)	C7C—C8C	1.286 (8)
C1—C2	1.385 (4)	C7C—H7C1	0.9900
C1—H1	0.9500	C7C—H7C2	0.9900
C2—C3	1.380 (4)	C8C—H8C1	0.9800
C2—H2	0.9500	C8C—H8C2	0.9800
C3—C4	1.386 (4)	C8C—H8C3	0.9800
C3—H3	0.9500	C1C'—C2C'	1.505 (19)
C4—C5	1.392 (4)	C1C'—H1C3	0.9900
C4—H4	0.9500	C1C'—H1C4	0.9900
C5—C6	1.469 (4)	C2C'—H2C4	0.9800
C6—C7	1.387 (3)	C2C'—H2C5	0.9800
C7—C8	1.379 (4)	C2C'—H2C6	0.9800
C7—H7	0.9500	C3C'—C4C'	1.522 (12)
C8—C9	1.383 (4)	C3C'—H3C3	0.9900
C8—H8	0.9500	C3C'—H3C4	0.9900
C9—C10	1.386 (4)	C4C'—H4C4	0.9800
C9—H9	0.9500	C4C'—H4C5	0.9800
C10—H10	0.9500	C4C'—H4C6	0.9800
N3—C11	1.147 (4)	C5C'—C6C'	1.504 (18)
N4—C12	1.155 (3)	C5C'—H5C3	0.9900
N5—C13	1.159 (3)	C5C'—H5C4	0.9900
N6—C14	1.151 (3)	C6C'—H6C4	0.9800
N1C—C3C	1.485 (4)	C6C'—H6C5	0.9800
N1C—C7C'	1.489 (6)	C6C'—H6C6	0.9800
N1C—C1C'	1.509 (6)	C7C'—C8C'	1.256 (11)
N1C—C5C'	1.521 (6)	C7C'—H7C3	0.9900
N1C—C7C	1.526 (4)	C7C'—H7C4	0.9900
N1C—C1C	1.528 (4)	C8C'—H8C4	0.9800
N1C—C5C	1.555 (4)	C8C'—H8C5	0.9800
N1C—C3C'	1.570 (7)	C8C'—H8C6	0.9800
C1C—C2C	1.506 (16)	N1S—C1S	1.136 (4)
C1C—H1C1	0.9900	C1S—C2S	1.443 (4)
C1C—H1C2	0.9900	C2S—H2S1	0.9800
C2C—H2C1	0.9800	C2S—H2S2	0.9800
C2C—H2C2	0.9800	C2S—H2S3	0.9800
C2C—H2C3	0.9800	O1W—H1W1	0.827 (18)
C3C—C4C	1.518 (9)	O1W—H2W1	0.821 (18)
C3C—H3C1	0.9900	O2W—H1W2	0.84 (2)
C3C—H3C2	0.9900	O2W—H2W2	0.84 (2)
			
C12—Co1—C13	87.23 (11)	H2C2—C2C—H2C3	109.5
C12—Co1—C11	89.91 (11)	N1C—C3C—C4C	114.4 (5)
C13—Co1—C11	88.80 (12)	N1C—C3C—H3C1	108.7
C12—Co1—C14	88.53 (10)	C4C—C3C—H3C1	108.7
C13—Co1—C14	90.20 (11)	N1C—C3C—H3C2	108.7
C11—Co1—C14	178.18 (10)	C4C—C3C—H3C2	108.7
C12—Co1—N1	95.18 (10)	H3C1—C3C—H3C2	107.6
C13—Co1—N1	177.20 (10)	C3C—C4C—H4C1	109.5
C11—Co1—N1	89.79 (11)	C3C—C4C—H4C2	109.5
C14—Co1—N1	91.28 (9)	H4C1—C4C—H4C2	109.5
C12—Co1—N2	177.28 (10)	C3C—C4C—H4C3	109.5
C13—Co1—N2	95.33 (10)	H4C1—C4C—H4C3	109.5
C11—Co1—N2	91.07 (10)	H4C2—C4C—H4C3	109.5
C14—Co1—N2	90.54 (9)	C6C—C5C—N1C	115.0 (7)
N1—Co1—N2	82.28 (8)	C6C—C5C—H5C1	108.5
C1—N1—C5	119.0 (2)	N1C—C5C—H5C1	108.5
C1—N1—Co1	126.39 (17)	C6C—C5C—H5C2	108.5
C5—N1—Co1	114.58 (17)	N1C—C5C—H5C2	108.5
C10—N2—C6	118.8 (2)	H5C1—C5C—H5C2	107.5
C10—N2—Co1	126.51 (17)	C5C—C6C—H6C1	109.5
C6—N2—Co1	114.64 (17)	C5C—C6C—H6C2	109.5
N1—C1—C2	122.0 (2)	H6C1—C6C—H6C2	109.5
N1—C1—H1	119.0	C5C—C6C—H6C3	109.5
C2—C1—H1	119.0	H6C1—C6C—H6C3	109.5
C3—C2—C1	119.1 (3)	H6C2—C6C—H6C3	109.5
C3—C2—H2	120.4	C8C—C7C—N1C	132.3 (5)
C1—C2—H2	120.4	C8C—C7C—H7C1	104.2
C2—C3—C4	119.4 (3)	N1C—C7C—H7C1	104.2
C2—C3—H3	120.3	C8C—C7C—H7C2	104.2
C4—C3—H3	120.3	N1C—C7C—H7C2	104.2
C3—C4—C5	119.1 (3)	H7C1—C7C—H7C2	105.5
C3—C4—H4	120.5	C7C—C8C—H8C1	109.5
C5—C4—H4	120.5	C7C—C8C—H8C2	109.5
N1—C5—C4	121.4 (2)	H8C1—C8C—H8C2	109.5
N1—C5—C6	114.4 (2)	C7C—C8C—H8C3	109.5
C4—C5—C6	124.2 (2)	H8C1—C8C—H8C3	109.5
N2—C6—C7	121.4 (2)	H8C2—C8C—H8C3	109.5
N2—C6—C5	114.1 (2)	C2C'—C1C'—N1C	116.7 (14)
C7—C6—C5	124.5 (2)	C2C'—C1C'—H1C3	108.1
C8—C7—C6	119.4 (2)	N1C—C1C'—H1C3	108.1
C8—C7—H7	120.3	C2C'—C1C'—H1C4	108.1
C6—C7—H7	120.3	N1C—C1C'—H1C4	108.1
C7—C8—C9	119.2 (2)	H1C3—C1C'—H1C4	107.3
C7—C8—H8	120.4	C1C'—C2C'—H2C4	109.5
C9—C8—H8	120.4	C1C'—C2C'—H2C5	109.5
C8—C9—C10	118.7 (3)	H2C4—C2C'—H2C5	109.5
C8—C9—H9	120.7	C1C'—C2C'—H2C6	109.5
C10—C9—H9	120.7	H2C4—C2C'—H2C6	109.5
N2—C10—C9	122.5 (2)	H2C5—C2C'—H2C6	109.5
N2—C10—H10	118.8	C4C'—C3C'—N1C	114.8 (8)
C9—C10—H10	118.8	C4C'—C3C'—H3C3	108.6
N3—C11—Co1	179.5 (3)	N1C—C3C'—H3C3	108.6
N4—C12—Co1	179.4 (2)	C4C'—C3C'—H3C4	108.6
N5—C13—Co1	179.0 (3)	N1C—C3C'—H3C4	108.6
N6—C14—Co1	178.5 (2)	H3C3—C3C'—H3C4	107.5
C3C—N1C—C7C'	157.9 (4)	C3C'—C4C'—H4C4	109.5
C3C—N1C—C1C'	87.4 (3)	C3C'—C4C'—H4C5	109.5
C7C'—N1C—C1C'	112.1 (4)	H4C4—C4C'—H4C5	109.5
C3C—N1C—C5C'	66.9 (3)	C3C'—C4C'—H4C6	109.5
C7C'—N1C—C5C'	113.2 (4)	H4C4—C4C'—H4C6	109.5
C1C'—N1C—C5C'	109.2 (4)	H4C5—C4C'—H4C6	109.5
C3C—N1C—C7C	110.9 (3)	C6C'—C5C'—N1C	113.9 (12)
C7C'—N1C—C7C	49.2 (3)	C6C'—C5C'—H5C3	108.8
C1C'—N1C—C7C	161.2 (4)	N1C—C5C'—H5C3	108.8
C5C'—N1C—C7C	82.9 (3)	C6C'—C5C'—H5C4	108.8
C3C—N1C—C1C	113.1 (3)	N1C—C5C'—H5C4	108.8
C7C'—N1C—C1C	71.8 (3)	H5C3—C5C'—H5C4	107.7
C1C'—N1C—C1C	58.8 (3)	C5C'—C6C'—H6C4	109.5
C5C'—N1C—C1C	167.6 (3)	C5C'—C6C'—H6C5	109.5
C7C—N1C—C1C	107.9 (3)	H6C4—C6C'—H6C5	109.5
C3C—N1C—C5C	108.6 (3)	C5C'—C6C'—H6C6	109.5
C7C'—N1C—C5C	89.4 (3)	H6C4—C6C'—H6C6	109.5
C1C'—N1C—C5C	66.5 (3)	H6C5—C6C'—H6C6	109.5
C5C'—N1C—C5C	62.7 (3)	C8C'—C7C'—N1C	138.0 (9)
C7C—N1C—C5C	109.4 (3)	C8C'—C7C'—H7C3	102.6
C1C—N1C—C5C	106.9 (3)	N1C—C7C'—H7C3	102.6
C3C—N1C—C3C'	54.2 (3)	C8C'—C7C'—H7C4	102.6
C7C'—N1C—C3C'	107.4 (4)	N1C—C7C'—H7C4	102.6
C1C'—N1C—C3C'	109.0 (4)	H7C3—C7C'—H7C4	105.0
C5C'—N1C—C3C'	105.7 (4)	C7C'—C8C'—H8C4	109.5
C7C—N1C—C3C'	80.1 (3)	C7C'—C8C'—H8C5	109.5
C1C—N1C—C3C'	82.7 (3)	H8C4—C8C'—H8C5	109.5
C5C—N1C—C3C'	162.8 (3)	C7C'—C8C'—H8C6	109.5
C2C—C1C—N1C	115.4 (8)	H8C4—C8C'—H8C6	109.5
C2C—C1C—H1C1	108.4	H8C5—C8C'—H8C6	109.5
N1C—C1C—H1C1	108.4	N1S—C1S—C2S	179.2 (4)
C2C—C1C—H1C2	108.4	C1S—C2S—H2S1	109.5
N1C—C1C—H1C2	108.4	C1S—C2S—H2S2	109.5
H1C1—C1C—H1C2	107.5	H2S1—C2S—H2S2	109.5
C1C—C2C—H2C1	109.5	C1S—C2S—H2S3	109.5
C1C—C2C—H2C2	109.5	H2S1—C2S—H2S3	109.5
H2C1—C2C—H2C2	109.5	H2S2—C2S—H2S3	109.5
C1C—C2C—H2C3	109.5	H1W1—O1W—H2W1	108 (3)
H2C1—C2C—H2C3	109.5	H1W2—O2W—H2W2	105 (4)

### Hydrogen-bond geometry (Å, °)

**Table:**

*D*—H···*A*	*D*—H	H···*A*	*D*···*A*	*D*—H···*A*
O1W—H1W1···N5^i^	0.83 (2)	2.10 (2)	2.925 (3)	176 (3)
O1W—H2W1···N4	0.82 (2)	2.08 (2)	2.899 (3)	174 (3)
O2W—H1W2···N3	0.84 (2)	1.83 (2)	2.665 (5)	172 (7)
O2W—H2W2···N3^ii^	0.84 (2)	1.92 (2)	2.740 (5)	165 (7)

Symmetry codes: (i) *x*+1/2, −*y*+1/2, *z*+1/2; (ii) −*x*+2, −*y*, −*z*.

## References

[bb1] Darensbourg, J. & Phelps, A. L. (2004). *Inorg. Chim. Acta*, **357**, 1603–1607.

[bb2] Garde, R., Villain, F. & Verdaguer, M. (2002). *J. Am. Chem. Soc.* **124**, 10531–10538. 10.1021/ja020528z 12197755

[bb3] Holmes, S. M. & Girolami, G. S. (1999). *J. Am. Chem. Soc.* **121**, 5593–5594.

[bb4] Lescoueezec, R., Lloret, F., Julve, M., Vaissermann, J. & Verdaguer, M. (2002). *Inorg. Chem.* **41**, 818–826. 10.1021/ic0107882 11849082

[bb5] Lyubartseva, G. & Parkin, S. (2009). *Acta Cryst.* E**65**, m1530. 10.1107/S1600536809046108 PMC2971905 21578573

[bb6] Mallah, T., Thibault, S., Verdaguer, M. & Veillet, P. (1993). *Science*, **262**, 1554–1557. 10.1126/science.262.5139.1554 17829385

[bb7] Nonius (1998). *COLLECT* Nonius BV, Delft, The Netherlands.

[bb8] Otwinowski, Z. & Minor, W. (1997). *Methods in Enzymology*, Vol. 276, *Macromolecular Crystallography*, Part A, edited by C. W. Carter Jr & R. M. Sweet, pp. 307–326. New York: Academic Press.

[bb9] Sheldrick, G. M. (2008). *Acta Cryst* A**64**, 112–122. 10.1107/S0108767307043930 18156677

[bb10] Sokol, J. J., Hee, A. G. & Long, J. R. (2002). *J. Am. Chem. Soc.* **124**, 7656–7657. 10.1021/ja0263846 12083909

[bb11] Szalda, D. J., Creutz, C., Mahajan, D. & Sutin, N. (1983). *Inorg. Chem.* **22**, 2372–2379.

[bb12] Toma, L., Lescouezec, R., Uriel, S., Llusar, R., Ruiz-Perez, C., Vaissermann, J., Lloret, F. & Julve, M. (2007). *J. Chem. Soc. Dalton Trans.* pp. 3690–3698. 10.1039/b705084b 17700833

[bb13] Toma, L., Lescouezec, R., Vaissermann, J., Delgado, F. S., Ruiz-Perez, C., Carrasco, R., Cano, J., Lloret, F. & Julve, M. (2004). *Chem. Eur. J.* **10**, 6130–6145. 10.1002/chem.200400611 15515063

